# Changes in Kidney Function Following Living Donor Nephrectomy: A Retrospective, Single-Center, Descriptive Study

**DOI:** 10.7759/cureus.90473

**Published:** 2025-08-19

**Authors:** Christy Rajeevkumar Ratnakumar, Mohamed Nazar Abdul Latiff, Gayathri Rajeevkumar

**Affiliations:** 1 Nephrology, National Hospital of Sri Lanka, Colombo, LKA; 2 South West Thames Renal and Transplantation Unit, St Helier University Hospital, London, GBR; 3 General Internal Medicine, National Hospital of SriLanka, Colombo, LKA

**Keywords:** a descriptive cross-sectional study, chronic kidney disease, kidney function, kidney transplant, live donor nephrectomy

## Abstract

Background

Kidney transplantation is the best possible option for patients reaching end-stage renal disease. Despite the excellent outcomes for recipients in terms of graft survival, the evidence regarding outcomes for donors is conflicting, and there is uncertainty regarding the long-term complications of kidney donation. Thus, our study aimed to assess the change in kidney function following donor nephrectomy and identify the associated comorbidities in a Sri Lankan cohort.

Methodology

This descriptive retrospective study was conducted at the donor clinic of Unit 1 at the National Hospital of Sri Lanka from July 2021 to November 2021. A total of 102 donors who had undergone donor nephrectomy since 2005 were included.

Results

The mean duration of the study population since donation was six years, with a standard deviation of ±3.20. The minimum duration since donation was one year, and the maximum was 14 years. The median interquartile range (IQR) preoperative glomerular filtration rate (GFR) was 94.50 (19) ml/min; the GFR on discharge was 66.50 (29) ml/min; and the current median GFR was 73.86 (23.31) ml/min. These changes in the GFR were statistically insignificant, with a p-value of 0.062. The preoperative estimated glomerular filtration rates (eGFRs) of all 102 donors (100%) exceeded 60 ml/min/1.73 m^2^, while the current values showed that 72 members of the study group (70.6%) had values exceeding 60 ml/min/1.73 m^2^, and 30 members of the study group (29.4%) had values of less than 60 ml/min/1.73 m^2^. The difference in eGFR values between the preoperative and current states was <15 in 24 patients (23.5%). eGFR values between 15 and 30 were observed in 49 members of the study population (48%), while eGFR values of >30 were reported in 29 patients (28.4%).

The median preoperative (IQR) urine protein creatinine ratio (UPCR) was 0.00 (0.05) mg/mmol, and it changed to 0.01 (0.14) mg/mmol. Serum creatinine (SCr) levels increased in 89 members of the study population (98.9%) while remaining unchanged in one member (1.1%). Both UPCR and SCr values showed statistically insignificant changes following donation (p-values of 0.332 and 0.119, respectively). In terms of comorbidities, 18.8 percent of the patients (n=19) had hypertension before surgery, and the prevalence of hypertension increased to 31.0 percent (n=31) after surgery. Preoperatively, obesity was found in 11.1% (n=11) and, postoperatively, 12.1% of the cases (n=12) were associated with obesity.

Among the data on the association between the GFR and the factors affecting change in the kidney function, a statistically significant association was observed between age and GFR values, with a p-value of <0.001. However, neither gender, smoking, body mass index, income, duration since donation, the frequency of follow-up visits, comorbidities such as hypertension, nor the type of donor showed any association with GFR. Likewise, the development of proteinuria was not associated with any of these factors. Notably, renal functions (GFR, SCr, and UPCR) and duration after donation showed no statistically significant associations.

Conclusion

The GFR tended to drop immediately postoperatively, but no sustained further drop in the GFR over time was observed. Similarly, no statistically significant risk of developing proteinuria with kidney donation was observed. In our findings, older age was the only factor related to an increased likelihood of a decrease in the GFR following donor nephrectomy.

## Introduction

Chronic kidney disease (CKD) affects nearly 10% of the global population. In Sri Lanka, the point prevalence of CKD varies across districts from 3% to 20% [1]. Beyond conventional risk factors such as diabetes and hypertension, the growing prevalence of CKD of unknown etiology in Sri Lanka has recently increased the burden of the disease.

For patients who reach end-stage renal failure, kidney transplantation is the best option [2]. Live donor kidney transplantation is preferred over deceased donor transplantation because it is associated with improved graft survival [3]. Most donors are related genetically to the recipients, such as children, parents, or siblings [4].

The first successful kidney transplantation in Sri Lanka was performed in 1988. Following the establishment of a national deceased donor kidney transplant program in Sri Lanka, there has been a surge in transplant operations. The National Hospital of Sri Lanka (NHSL) carries out most of the renal transplants in the country. The demand for living donors is high in our center, as it is in other centers [5]. The donor shortage is mainly attributable to the perceived harms to donors in terms of morbidity and mortality.

Kidney transplantation carries a small perioperative mortality risk of 0.03% for donors [3]. Beyond the immediate risk, the long-term risk of developing end-stage renal failure and increased cardiovascular risk is of concern. Theoretically, a 50% reduction in the glomerular filtration rate (GFR) is expected after nephrectomy, but studies report only a 25% to 40% reduction. This discrepancy is attributable to compensatory hyperfiltration by the remaining kidney. Several studies have shown that this hyperfiltration leads to the development of proteinuria, but it has not been determined yet whether the proteinuria leads to a progressive deterioration in renal function [6,7]. In any case, the long-term implications of kidney donation are uncertain. The overall life expectancy of kidney donors is perceived to be comparable to that of the normal population. Some studies have shown better survival, while others have shown an increased risk of end-stage renal failure [3].

Several international studies have evaluated the donor outcomes and complications of kidney transplantation in donors [4,5,7]. The evidence is conflicting, mainly because of the lack of proper controls, the use of small samples in studies, and discontinuity in follow-up. To our knowledge, no study conducted in Sri Lanka to date has evaluated the outcomes for donors after kidney transplantation.

Garg et al. carried out a systematic review and a meta-analysis involving 48 studies from 27 countries to evaluate the development of proteinuria and the deterioration in renal function in living kidney donors at an average of seven years after transplant [6]. These researchers observed significant heterogeneity in proteinuria across the studies that they analyzed, with the incidence ranging from 5% to more than 20%, and concluded that kidney donation leads to a small increase in proteinuria (averaging 154 mg/dl) that becomes more pronounced over time. A meta-regression analysis showed that age at donation, gender, and blood pressure at the time of donation were not associated with the development of proteinuria and that an initial decrement occurred in the GFR, with 12% of donors developing a GFR of less than 60 ml/min and 0.2% developing a GFR of less than 30 ml/min. An initial decrement in the GFR after donation was not accompanied by accelerated loss. Though the pre- and post-donation GFRs were low among females and older individuals, no statistically significant changes were observed in the GFRs after donation. Further, neither body mass index (BMI), serum uric acid, cholesterol level, ethnicity, nor blood pressure was related to decreases in GFRs.

A recent retrospective cohort study in Canada analyzed GFR trends in 604 donors by comparing them with 2,414 healthy matched non-donors [7]. The researchers found that the estimated glomerular filtration rate (eGFR) increased from six weeks after donation onwards. The compensation was greatest in the first two years and started to plateau in the fifth year. The eGFR after donation varied by gender, being higher among female donors. No association was observed with age at donation, pre-donation blood pressure, pre-donation eGFR category, or socioeconomic status.

Several studies have evaluated the risk of developing hypertension after renal transplantation. A meta-analysis by Boudville et al. of 48 studies from 28 countries involving 5,145 donors conducted in 2006 concluded that kidney donors may experience a 5 mmHg increase in blood pressure within five to 10 years after donation over the increase anticipated with normal aging [8]. By contrast, a study by Ibrahim et al. found no increased risk of hypertension associated with kidney donation [9]. Various studies have presented conflicting evidence regarding the association between pre-existing hypertension and post-donation GFR [8-10]. Several studies have reported no association between pre-donation hypertension and post-donation deterioration in the GFR [6,7,9-11].

Other studies have addressed the risk of end-stage renal failure and cardiovascular risk, which are of long-term concern for kidney donors. Ibrahim et al. evaluated the risk of end-stage renal disease (ESRD) in 3,698 kidney donors and found that 11 of them developed ESRD, 14.5% had GFRs of less than 60 ml/min, 32.1% had hypertension, and 12.7% had albuminuria [5]. These results indicated that the occurrence of proteinuria and hypertension, the life span, the risk of ESRD, and the quality of life for donors were similar to the general population. The findings of this study that low GFRs and hypertension were associated with older age and higher BMI but not with the time since donation support the view that the factors linked to a reduced GFR in donors are the same as those observed in the general population.

A study conducted in Oslo University Hospital in 2014 evaluated 1,901 donors and compared them with matched controls [12]. During the observation period (median 15 years), a significant increase was observed in cardiovascular mortality among donors: the hazard ratio (HR) was 1.40, the 95% confidence interval (CI) was 1.03-1.91, and p=0.03. The analysis demonstrated a significant increase in ESRD in kidney donors, with an HR of 11.38 (4.37-29.63, p<0.001). Immunological diseases contributed to most of the occurrences of renal failure since most were immediate family members. The researchers postulated that the increased incidence of ESRD in the cohort of kidney donors was related to hereditary factors rather than nephrectomy. They also observed an increased all-cause mortality, with an HR of 1.30 (95% CI, 1.11-1.52, p=0.001).

Several studies have shown that donor survival rates are similar to the general population, but one study, by Fehrman-Ekhol et al., evaluated 459 donors and found a 29% higher survival rate in donors [13]. This finding may be due to the fact that healthy people are accepted as donors through proper donor screening. This study also showed that donors had mortality patterns comparable to those of the normal aging population and that nephrectomy was not associated with mortality.

Garg et al. conducted a retrospective population-based matched cohort study following a total of 2,028 donors and 2,028 matched non-donors over 6.5 years [6]. The risks of death and cardiovascular events were lower in the donors than in the non-donors (2.8 compared with 4.1 events per 1,000 person-years, respectively; HR 0.66, 95% CI 0.48-0.90). Older age and lower income were associated with high risk in both groups.

Janki et al. conducted a prospective cohort study with 100 donors [14]. Interestingly, they reported a significant decrease in quality of life and physical function and a high incidence of body pain and fatigue (p<0.001). Nevertheless, donor outcomes were excellent 10 years after donation, with kidney function appearing stable and hypertension seemingly no more frequent than in the general population. At 10 years median follow-up, only nine of the donors had died, all from causes unrelated to donation.

The present study adds to the available evidence in support of the safety of the practice for carefully selected donors.

## Materials and methods

This single-center, descriptive retrospective study was carried out in the Donor Clinic of Nephrology Unit 1 at the NHSL in Colombo. All of the patients who had undergone donor nephrectomy since 2005 were included in the study. They were recruited from the donor registry of Nephrology Unit 1. Individuals who had donated within the previous year were excluded. All of the patients fulfilling the inclusion criteria were included in the study. The study was conducted from July to November 2021. Ethical approval was granted by the Ethical Review Committee of the NHSL. The participants supplied the required information by completing a structured questionnaire using direct questioning, past records, and laboratory reports. They were interviewed in person. Their body weight, height, and blood pressure were measured with standard procedures. The laboratory investigations were conducted in the NHSL. Serum creatinine (SCr) values were assessed using the Abbott Architect c8000 analyzer (Abbott Laboratories, USA) and the enzymatic method. GFRs were calculated using the 2009 Chronic Kidney Disease Epidemiology Collaboration (CKD-EPI) creatinine equation, and proteinuria was assessed using a Konelab 30 analyzer (Thermo Fisher Scientific, Finland) and the pyrogallol red method. The data were entered into a pre-tested, interviewer-administered questionnaire and later into a computer database. The data collection was done by qualified doctors from the Sri Lanka Medical Council and the principal investigator. The data collected were presented as median and IQR. The CI was 95%, and p<0.05 indicated statistical significance.

In this study, descriptive statistics were used to summarize the demographic variables and baseline characteristics of the sample population. The continuous variables, such as age, duration of follow-up, and hospital stay, were expressed as mean ± standard deviation, while the categorical variables, including gender, marital status, education level, donor relationship, and smoking status, were presented as frequencies and percentages. Medians and IQRs were reported for key clinical measures such as kidney function markers, including the GFR, urine protein creatinine ratio (UPCR), blood pressure, and BMI.

Visual representation of the data was performed using bar charts and pie charts to illustrate the patients’ demographic characteristics (gender, marital status, ethnicity, education, employment, and income), the distribution of preoperative and postoperative comorbidities (hypertension, overweight, and obesity), and recipient outcomes (survival or death). The Spearman correlation test served to assess the relationships among the variables, in particular, the correlation between the duration after donation and the renal function parameters (SCr, UPCR, and GFR), as well as the blood pressure values. The associations between the categorical variables and kidney function outcomes were examined using Chi-square tests or Fisher’s exact tests, depending on the sample size and the expected cell counts. These analyses assessed the factors that influence GFR categories (<60 compared with >60 ml/min) and UPCR categories (<0.3 compared with >0.3) by considering variables such as age, gender, smoking status, income, donor type, BMI, hypertension, clinic visits, distance to hospital from home, and time after donation.

A logistic regression model was developed to identify predictors of recipient mortality following donor nephrectomy. The model included gender, donor relationship, and current SCr as independent variables. The model coefficients, statistical significance, odds ratios, and 95% CIs were reported. The adequacy of the model was confirmed using the Hosmer-Lemeshow goodness-of-fit test, which indicated an appropriate fit.

Throughout the analysis, p-values were used to determine the statistical significance of the observed differences or associations, including the comparison of preoperative and current renal function values.

## Results

This section highlights the donors’ socioeconomic backgrounds and relationships to the patients, as well as changes in and factors affecting kidney function following surgery. Also discussed are postoperative complications and outcomes, new comorbidities following surgery, and their association with kidney function, and the associations of the GFR and the UPCR with various factors. The total sample size was 102 donors. The mean age of the patients in the study was 49 ± 9.845 years.

**Figure 1:**
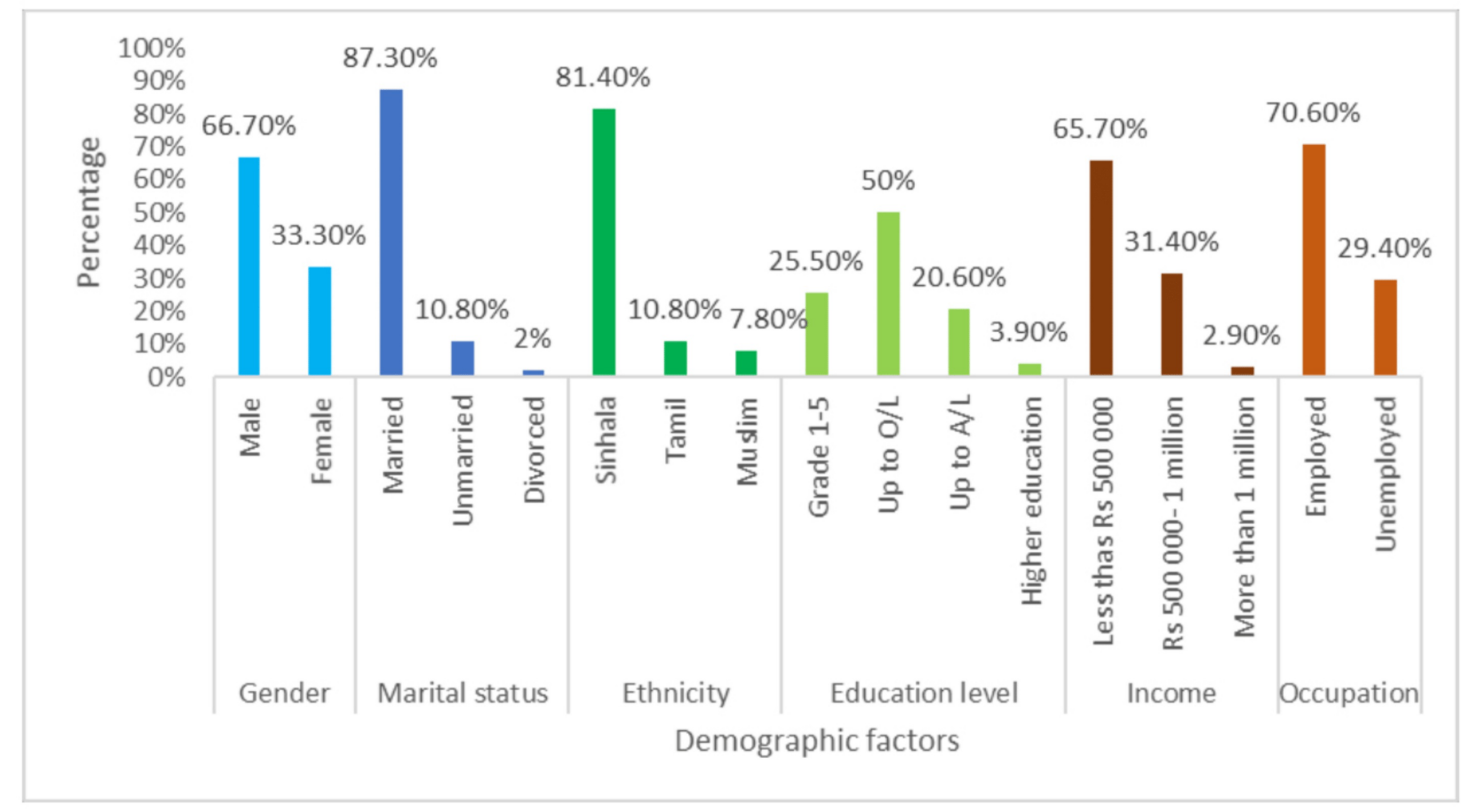
Demographic details of the sample O/L, ordinary level; A/L, advanced level.

The bar chart in Figure 1 illustrates the demographic characteristics of the sample. Sixty-eight of the participants (66.70%) were male, so there was a male predominance in the sample population. Most were married (89; 87.30%), 11 (10.80%) were unmarried, and the other 2 (2%) were divorced. Most (83; 81.40%) were Sinhalese, 11 (10.80%) were Tamils, and 8 (7.80%) were Muslims. Half (51; 50%) were educated up to the ordinary level, and 4 (3.90%) had pursued higher education. Most (72; 70.60%) were employed, but 67 (65.70%) were earning less than Rs. 500,000 annually, 32 (31.40%) were earning between Rs. 500,000 and Rs. 1 million, and 3 (2.90%) were earning more than Rs. 1 million.

According to the data presented in Table 1, 38 of the donors (36.3%) were related to the recipients, and the remaining 64 (63.7%) were unrelated. Among the donors, parents were the most frequent donors (27; 26.5%), followed by spouses and friends (22; 21.6%), altruistic donors (13; 12.7%), siblings (11; 10.8%), and other relatives of the patients (7; 6.9%).

**Table 1:** The donors’ relationship with the patients

Relationship of the donor	Frequency (%)
Type of donor	
Related	38 (36.3)
Non-related	64 (63.7)
Relationship to the donor	
Sibling	11 (10.8)
Parent	27 (26.5)
Spouse	22 (21.6)
Friend	22 (21.6)
Altruistic	13 (12.7)
Others	7 (6.9)

The mean duration of the study population (Table 2) since the donation was six years, with a standard deviation of ±3.20 years and a range of one to 14 years. Most of the donors (63; 61.8%) were aware of the etiology of the recipients’ CKD, and the rest (39; 38.2%) were unaware. Only 2 donors (2%) followed up with three clinical visits after donation, while 55 (53.9%) did not follow up at all, 7 (6.9%) followed up with two visits, and 38 (37.3%) followed up with a single visit. Fourteen (13.7%) of the donors were smokers, while 88 (86.3%) were nonsmokers. The postoperative complications noted in the study sample in the table above suggest that only 1 (1%) suffered from infection during the postoperative period. The mean duration of the donors’ hospital stays was 3.72 days, with a standard deviation of 2.17 days.

**Table 2:** Factors that may be associated with changes in kidney function following donor nephrectomy CKD: chronic kidney disease, SD: standard deviation.

Factors	Frequency (%)
Awareness of the etiology of CKD in the recipient	
Aware	63 (61.8)
Unaware	39 (38.2)
Duration after donation (years)	
Minimum	1 year
Maximum	14 years
Mean ± SD	6 ± 3.20 years
Number of clinic visits after donation	
0	55 (53.9)
1	38 (37.3)
2	7 (6.9)
3	2 (2.0)
Smoking status	
Yes	14 (13.7)
No	88 (86.3)
Duration of hospital stay (days) (Mean ± SD)	3.72 ± 2.168 days
Postoperative infections	
Yes	1 (1.0)
No	101 (99.0)

**Figure 2:**
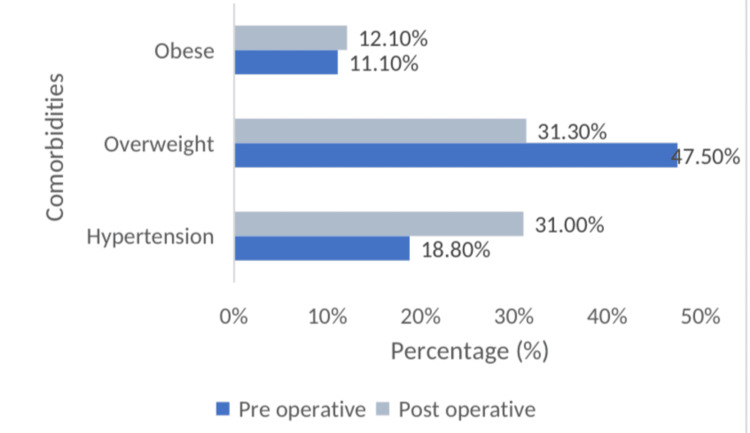
Distribution of comorbidities (preoperative period and postoperative period)

Evaluation of the data regarding the donors’ preoperative and postoperative status (Figure 2) revealed that 19 of them (18.8%) had hypertension before the operation, while 31 (31.0%) had the condition after the operation. Forty-seven (47.5%) were overweight before the operation, while 31 (31.3%) were overweight after the operation, and 11 (11.1%) were obese before the operation, while 12 (12.1%) were obese after the operation.

Evaluation of the data on the renal function of the donors (Table 3) indicated that the median (IQR) of the pre-GFR, the current GFR, and the difference in the GFR values were 94.5 (19) ml/min, 73.86 (23.31) ml/min, and 20.40 (33.32) ml/min, respectively. The median (IQR) of the pre-GFR, the current GFR, and the difference in the UPCR values were 0.00 (0.05) mg/mmol, 0.1 (0.14) mg/mmol, and -0.08 (0.09) mg/mmol, respectively. The median (IQR) values for systolic blood pressure for the pre-GFR, the current GFR, and the difference in the GFR values were 123 (29) mm Hg, 120.80 (29) mm Hg, and -1.00 (121) mm Hg, respectively. The median (IQR) for diastolic blood pressure of the pre-evaluation, the current evaluation, and the difference in the values were 70 (10) mm Hg, 80 (10) mm Hg, and -60 (80) mm Hg, respectively. The median (IQR) for the BMI of the pre-evaluation, the current evaluation, and the differences in the values were 25.68 (6.30) kg/m2, 24.10 (7.27) kg/m2, and -21 (22) kg/m2, respectively. 

**Table 3:** Descriptive statistics of the donors’ renal function GFR: glomerular filtration rate, ml/min; IQR: interquartile range; UPCR: urine protein: creatinine ratio, mg/mmol; SBP: systolic blood pressure, mm Hg; DBP: diastolic blood pressure, mm Hg; BMI: body mass index.

Renal function	Descriptive statistics			
	Median	IQR	Minimum	Maximum
GFR				
Pre	94.5	19	75	123
Current	73.86	23.31	39.16	110.25
Difference (pre-current)	20.4	33.32	-110.25	74
UPCR				
Pre	0	0.05	0	0.22
Current	0.1	0.14	0	1.39
Difference (pre-current)	-0.08	0.09	-1.39	0.17
SBP descriptive of comorbidities				
Pre	123	29	90	140
Current	120.8	29	90	220
Difference (pre-current)	-1	121	-180	19
DBP				
Pre	70	10	60	90
Current	80	10	60	110
Difference (pre-current)	-60	80	-110	10
BMI				
Pre	25.68	6.3	14.5	31.3
Current	24.1	7.27	12.5	39
Difference (pre-current)	-21	22	-39	8.1

We assessed the association between the median values of preoperative and current renal functions. The results are presented in Table 4. The median (IQR) values for the preoperative and current SCr levels were 0.84 (0.22) mg/dl and 1.10 (0.34) mg/dl, respectively. The p-values for the difference between the preoperative and current SCr, GFR, UPCR, systolic blood pressure (SBP), and BMI were 0.119, 0.062, 0.332, 0.233, and 0.089, respectively. None of the preoperative or current values of the renal functions had a statistically significant association. Consideration of the data on the renal function of the donors suggests a statistically insignificant change in the GFR value, with a p-value of 0.062. The GFR value tended to drop immediately postoperatively, but there was no sustained further drop in GFR over time.

**Table 4:** Renal function of the donors IQR: interquartile range; SCr: serum creatinine, mg/dl; GFR: glomerular filtration rate, ml/min; UPCR: urine protein creatinine ratio, mg/mmol; SBP: systolic blood pressure; BMI: body mass index.

	Median, IQR				
Renal function	Preoperative period	On discharge	On first clinic visit	Current	p-value
SCr	0.84 (0.22)	1.2 (0.41)	1.10 (0.50)	1.10 (0.34)	0.119
GFR	94.50 (19)	66.50 (29)	63 (15.04)	73.86 (23.31)	0.062
						UPCR	0.00 (0.05)	-	-	0.1 (0.14)	0.332
SBP	123 (29)	-	-	120.80 (29)	0.233						
BMI	25.68 (6.30)	-	-	24.10 (7.27)	0.089

**Figure 3:**
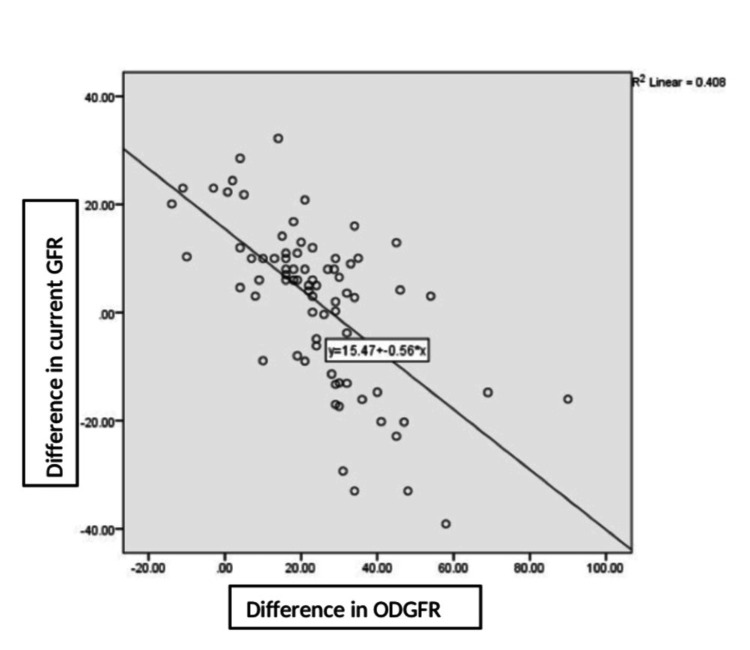
Scatter plot showing the relationship of the initial drop in the GFR to the further drop over time GFR: glomerular filtration rate, ml/min; ODGFR: on-discharge glomerular filtration; difference in the current GFR: preoperative GFR-current GFR; difference in the GFR on discharge: preoperative GFR-GFR on discharge.

A significant association was observed between the initial drop in the GFR (i.e., the difference between the preoperative GFR and the GFR on discharge) and the further drop in the GFR (i.e., the difference between the preoperative GFR and the current GFR), with a p-value of ≤0.001 (Figure 3). Though the SCr levels showed a slight change postoperatively, the change was statistically insignificant, with a p-value of 0.119. The changes in the UPCR values were also statistically insignificant, with a p-value of 0.332.

**Figure 4:**
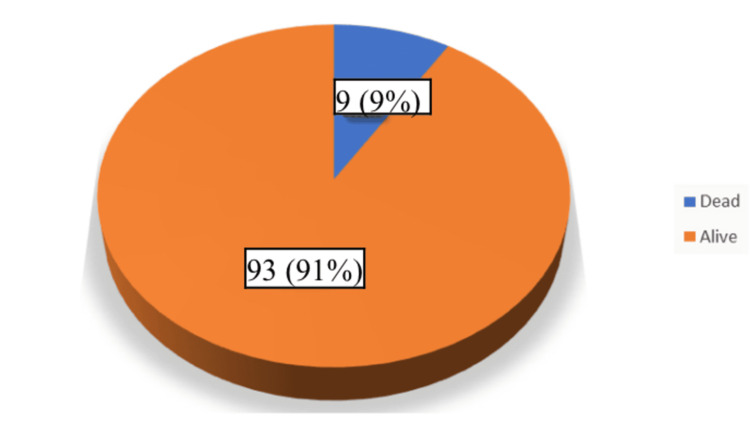
Outcome of the recipients

Overall, 9 (9%) of the recipients died following live donor nephrectomy (Figure 4).

The preoperative eGFR was >60 ml/min/1.73 m2inall 102 (100%) of the patients (Table 5), but the current values changed, with 72 (70.6 %) of them having values>60 ml/min/1.73 m2 and 30 (29.4%) having values of <60 ml/min/1.73 m2. Eighty (92%) of them had SCr levels of <1.1 mg/dl, but currently only 45 (44.1%) had values of <1.1 mg/dl. Levels of serum UPCR <0.3 were observed in almost all of the patients preoperatively, while currently, 3 (3.1%) of them had values>0.3, and the remaining 93 (96.9%) had serum UPCR levels remaining at<0.3.

**Table 5:** Renal function changes following donor nephrectomy eGFR: estimated glomerular filtration rate, ml/min/1.73 m2; SCr: serum creatinine, mg/dl; UPCR: urine protein : creatinine ratio.

Renal functions	Percentage (%) of donors in the preoperative period (n=102)	Percentage (%) of donors on discharge	Percentage (%) of donors on current status
		(n=75)	(n=102)
eGFR			
>60	102 (100.0)	50 (66.7)	72 (70.6)
<60	0 (0.0)	25 (33.3)	30 (29.4)
SCr			
>1.1 mg/dl	7 (8.0)	51 (68.0)	57 (55.9)
<1.1 mg/dl	80 (92.0)	24 (32.0)	45 (44.1)
Serum UPCR level			
Less than 0.3	90 (100.0)	-	93 (96.9)
More than 0.3	0 (0.0)	-	3 (3.1)

The difference in the eGFR values between the preoperative and current states was <15 in 24 patients (23.5%) (Table 6). Differences between 15 and 30 were observed in 49 patients (48%), and changes in the eGFR of <30 were observed in 29 patients (28.4%). The SCr level increased in 89 patients (98.9%) and was unchanged in 1 patient (1.1%).

**Table 6:** Preoperative and current kidney function changes eGFR: estimated glomerular filtration rate, ml/min/1.73 m2; SCr: serum creatinine.

Functions	Frequency (%)
Reduction in eGFR	
≤15	24 (23.5)
15-30	49 (48.0)
>30	29 (28.4)
Comparison of current and preoperative SCr levels	
Increased SCr level	89 (98.9%)
Unchanged SCr level	1 (1.1%)

The findings regarding the association between the GFR and the factors affecting the change in kidney function (Table 7) include a statistically significant association between age and GFR values, with a p-value of <0.001. Specifically, of the patients with GFR values of >60 ml/min, 45 (62.5%) were under 50 years of age, and 27 (39.5%) were over age 50. Thus, while the patients below the age of 50 years had higher GFR levels, most (76.7%) of the 23 individuals over 50 years of age had GFR values of <60 ml/min, while the remaining 23.3% of the patients who had GFR values of <60 ml/min included the 7 individuals below the age of 50. Regarding gender, 49 (68.1%) of the patients with GFR values of >60 ml/min were males, and the other 23 (31.9%) were females. Similarly, 19 of the patients with GFR values of <60 ml/min were male (63.3%), and 11 (36.7%) were female. The association of BMI with GFR had a p-value of 0.054; 46 (65.7%) of the patients with GFR values of >60 ml/min had a BMI of more than 23, and the remaining 24 (34.3%) had a BMI of less than 23. Sixteen of the patients (55.2%) with GFR values of <60 ml/min had a BMI of less than 23, and 13 of the patients (44.8%) with GFR values of <60 ml/min had a BMI of more than 23. Twelve (16.7%) of the patients with GFR values of >60 ml/min were smokers, and 2 (6.7%) of the patients with GFR values of <60 ml/min were smokers. Regarding income, 51 (70.8%) of the patients with GFR values of >60 ml/min had an income of <Rs. 500,000 annually, and 14 (46.7%) of the patients with GFR values of <60 ml/min had an annual income of >Rs. 500,000. The p-value for the type of donor was 0.395, with 28 (38.9%) of the related donors and 44 (61.1%) of the non-related donors having GFR values of >60 ml/min. Further, 18 (25%) of the donors with GFR values of >60 ml/min had had their nephrectomies performed within the previous five years, while 54 (75%) of the donors with GFR values of >60 ml/min had had their nephrectomies performed more than five years previously. The p-value for the factors of high BP and distance were 0.338 and 0.747, respectively.

**Table 7:** Association of GFR with factors affecting change in kidney function *Fisher’s exact value, Rs: rupees, km: kilometer, BMI: body mass index, GFR: glomerular filtration rate.

Risk factors	Current GFR (ml/min)		p-value
	Less than 60 (n=30)	More than 60 (n=72)	
Age			
Below 50 years	7 (23.3)	45 (62.5)	
Above 50 years	23 (76.7)	27 (39.5)	<0.001
Gender			
Male	19 (63.3)	49 (68.1)	0.645
Female	11 (36.7)	23 (31.9)	
Smoking status			
Yes	2 (6.7)	12 (16.7)	0.223*
No	28 (93.3)	60 (83.3)	
Duration after donation			
Less than 5 years	9 (30.0)	18 (25.0)	0.602
More than 5 years	21 (70.0)	54 (75.0)	
Income			
16 (53.3)	51 (70.8)	0.09
>Rs 500,000	14 (46.7)		21 (39.2)
Distance			
Less than 100km	19 (63.3)	48 (66.7)	0.747
More than 100km	11 (36.7)	24 (33.3)	
Number of clinic visits after			
0	14 (46.7)	41 (56.9)	
				1	12 (40.0)	26 (36.1)	
2	2 (6.7)	5 (6.9)	
3	2 (6.7)	0 (0.0)	-
Hypertension			
Yes	11 (37.9)	20 (38.2)	0.338
No	18 (62.1)	51 (71.8)	
BMI			
Less than 23	16 (55.2)	24 (34.3)	0.054
More than 23	13 (44.8)	46 (65.7)	
Type of donor			
Related	9 (30.0)	28 (38.9)	0.395
Not related	21 (70.0)	44 (61.1)	

The association of the UPCR (Table 8) with smoking had a p-value of 0.079, with 5 (62.5%) of the patients with UPCR values of >0.3 being nonsmokers and the remaining 3 (37.5%) of the patients with UPCR values of >0.3 being smokers. Similarly, of the patients with UPCR levels of <0.3, most (82, 88.2%) were nonsmokers, and the rest (11, 11.8%) were smokers. Regarding BMI, among the patients with UPCR levels of >0.3, 6 (75%) had a BMI of more than 23, and, among the individuals with UPCR levels of <0.3, 52 (57.8%) had a BMI of more than 23. Among the male patients, 7 (87.5%) had UPCR levels of >0.3, and 61 (65.6%) had UPCR levels of <0.3. Similarly, UPCR levels of >0.3 and <0 were mainly observed in the individuals without high BP (less than 0.3 -63, 69.2%; more than 0.3 - 5, 62.5%) and unrelated donors (<0.3 - 59, 63.4% ;>0.3 - 6, 75%). Six (75%) of the donors with UPCR levels of >0.3 and 61 (65.6%) of the donors with UPCR levels of <0.3 earned<Rs. 500,000. The p-values obtained for the association of UPCR level with age, duration after donation, and distance were similar (p=1.000). UPCR levels of <0.3 and >0.3 were distributed almost equally (50%) among the study population below and above the age of 50 years. Among the patients who had donated their organs within the previous five years, 25 (26.9%) had UPCR levels of <0.3, and 2 (25%) had UPCR levels of >0.3. Among the patients who had donated their organs more than five years previously, 68 (73.1%) had UPCR levels of <0.3, and 6 (75%) had UPCR levels of >0.3. Regarding distance from home to hospital, 61 (65.6%) of the patients who had a distance of less than 100 km had UPCR levels of >0.3, and 5 (62.5%) had UPCR levels of <0.3.

**Table 8:** Association of UPCR and risk factors *Fisher’s exact value, Rs: rupees, km: kilometer, BMI: body mass index, UPCR: urine protein: creatinine ratio.

Risk factors	Current UPCR		p-Value
	Less than 0.3 (n=93)	More than 0.3 (n=8)	
Age			
Below 50 years	47 (50.5)	4 (50.0)	1.000*
Above 50 years	46 (49.5)	4 (50.0)	
Gender			
Male	61 (65.6)	7 (87.5)	0.268*
Female	32 (34.4)	1 (12.5)	
Smoking status			
Yes	11 (11.8)	3 (37.5)	0.079*
No	82 (88.2)	5 (62.5)	
Duration after donation			
Less than 5 years	25 (26.9)	2 (25.0)	1.000*
More than 5 years	68 (73.1)	6 (75.0)	
Income			
< Rs 500,000	61 (65.6)	6 (75.0)	0.714*
>Rs 500,000	32 (34.4)	2 (25.0)	
Distance			
Less than 100km	61 (65.6)	5 (62.5)	1.000*
More than 100km	32 (34.4)	3 (37.5)	
Number of clinic visits after			
0	51 (54.8)	4 (50.0)	
				1	34 (36.6)	4 (50.0)	-
2	6 (6.5)	0 (0.0)	
3	2 (2.2)	0 (0.0)	
Blood pressure			
Yes	28 (30.8)	3 (37.5)	0.703*
No	63 (69.2)	5 (62.5)	
BMI			
Less than 23	38 (42.2)	2 (25.0)	0.466*
More than 23	52 (57.8)	6 (75.0)	
Type of donor			
Related	34 (36.6)	2 (25.0)	0.708*
Not related	59 (63.4)	6 (75.0)	

The association between the duration after donation and several renal functions was assessed (Table 9). The current SCr levels exhibited a negative correlation (R=-0.007) with duration after donation, but the association was not statistically significant (P=0.948). The UPCR and GFR current values correlated negatively with the duration after donation (R=-0.041 and R=-0.034, respectively). Both systolic and diastolic blood pressure values correlated positively with the duration after donation (R=0.113 and R=0.153, respectively). None of these findings had statistically significant associations with the duration after donation (p>0.05).

**Table 9:** Associations between duration after donation and renal function GFR: glomerular filtration rate, UPCR: urine protein: creatinine ratio.

Variable	R-value	P-value
Serum creatinine current status	-0.007	0.948
UPCR current status	-0.041	0.682
GFR current status	-0.034	0.734
Systolic blood pressure current	0.113	0.264
Diastolic blood pressure current	0.153	0.13

Even if the value of the independent variables (gender, relationship to the donor, and SCr) is zero, the odds value can be computed as -6.170. When the gender increases by one unit, the logic p (x) increases by 2.042 units. Similarly, when the spontaneous relationship to the donor and SCr current status increases by one unit, the logic p (x) value increases by 0.785 and 2.664, respectively (Table 10).

**Table 10:** Logistic regression for outcome (death or survival) Logit(px)=-6.170+2.042(Gender)+ 0.785(Relationship to the donor)+2.664 (serum creatinine current status), CI: confidence interval.

Variable	B	Significance	Exp(B)	CI(95%)	
				Lower	Upper
Gender	2.042	0.04	7.708	1.099	54.057
Relationship to the donor	0.785	0.031	2.192	1.073	4.475
Serum creatinine current status	2.664	0.032	14.357	1.258	163.883
Constant	-6.17	0.034			

Hosmer-Lemeshow test can be used to assess the goodness-of-fit test. The null hypothesis (H₀) states that the model fits the data well, meaning that there is no significant difference between observed and predicted values, while the alternative hypothesis (H₁) states that the model does not fit the data well.. The test yielded a p-value of 0.862, which is greater than the significance level of 0.05. Therefore, we do not reject the null hypothesis. This indicates that the logistic regression model fit the data adequately (Table 11).

**Table 11:** Test statistics

Chi square	Degree of freedom	Significance
3.95	8	0.862

## Discussion

The overall life expectancy of kidney donors is perceived to be comparable to that of the general population [13]. The risk of renal function deterioration and increased cardiovascular risk in donors is of concern but has yet to be explored comprehensively. Studies of renal deterioration following nephrectomy have yielded conflicting results in terms of the risk of progression to CKD and the associated risk factors. Researchers have found that certain characteristics, such as Black ethnicity, male gender, advanced age, hypertension, and obesity, can increase the risk of end-stage renal failure in certain individuals [15-17]. Renal function decreases immediately after nephrectomy but stabilizes within months and increases thereafter in most donors. On the other hand, a few donors have experienced further deterioration in renal function and a lower tolerance for the nephrectomy insult [18,19]. Sawhney et al. found that donors with a lower rate of post-recovery eGFR had a higher risk of developing end-stage renal failure [20]. Hence, poor recovery of renal function after nephrectomy could be an early indicator of a high risk of renal failure. The present study aimed to assess changes in kidney function following donor nephrectomy for live donor kidney transplant and to identify the comorbidities that developed following nephrectomy in a Sri Lankan cohort. An understanding of the post-donation changes in the GFR, SCr, and UPCR in relation to donor characteristics and the related risk factors for renal failure following nephrectomy may enhance physicians’ surveillance and early detection of the risk of developing end-stage kidney disease in donors.

We observed a decrease in GFRs during the immediate postoperative period but no further deterioration with follow-up. The median (IQR) of the difference between the preoperative and current GFR values was found to be 20.40 (33.32) ml/min. The change in GFRs was not statistically significant (p=0.062). This finding is consistent with the available evidence showing that, compared with the general population, donors do not experience an accelerated decrease in the GFR over time [21]. A similar pattern was observed in the SCr levels: while there was an elevation in levels to >1.1 mg/dl (n=51, 68.0%) at the time of discharge, the levels had decreased marginally at the current condition (n=57, 55.9%). We observed no statistically significant impact in terms of the development of proteinuria after nephrectomy (p=0.332). However, a meta-analysis of 48 studies by Garg et al. concluded that kidney donation leads to small increases in proteinuria (an average of 154 mg/dl), with significant heterogeneity among the studies [6].

Changes in kidney function compared with preoperative and present eGFR readings suggest that most of the donors (49, 48.0%) experienced a decrease of 15-30 in eGFRs, 29 (28.4%) experienced a decrease of >30, and 24 (53%) experienced a decrease of <15. Lam et al. demonstrated that six weeks after surgery, the eGFRs of the younger donors had decreased by approximately 33% while the eGFRs of the older donors had decreased by 35%-37%. Changes in the eGFR over time were influenced by the magnitude of the initial decline in the baseline eGFR within the first six weeks after surgery. Thus, the donors who experienced a >40% decline in the baseline eGFR after donation had, on average, a smaller increase in the eGFR over time compared with the donors who experienced a <30% decline in the baseline eGFR, though the results were not statistically significant [7]. Our study also showed a significant association between the initial decrease in the GFR (at discharge) and deterioration of the GFR over time, with a p-value of ≤0.001. Similarly, Massie et al. argued that the post-donation eGFR-but not the pre-donation eGFR-has predictive value for end-stage kidney disease [22].

Comparing the medians of the present SCr levels with the preoperative levels, we found that the SCr levels increased in 89 (98.9%) patients and remained unchanged in 1 (1.1%), and there was no statistically significant association (p=0.119). Chien et al. reported that higher baseline functioning of the preserved kidney prior to contralateral kidney excision was substantially associated with higher creatinine clearance from two days to 22 months after nephrectomy [23].

We found that most of the donors with an eGFR of less than 60 (n=23,76.7%) were over 50 years old. By contrast, eGFR ranges of >60 were observed in most of those under 50 (n=49,62.5%) (p=0.001). Thus, older age was associated with a higher risk of deterioration in renal function. Similarly, Chien et al. found lower renal function in older individuals [23], and this finding provides evidence for the relationship between GFR and advanced age in the general population. We found that gender did not appear to be statistically significant when it came to eGFR readings. By contrast, Lam et al. reported that male donors experienced a greater increase in eGFR over time than female donors [7]. Furthermore, in our study, neither the duration since donation nor the type of donor (related or not related) affected the reduction in the eGFR.

Our analysis of the duration after donation and renal functions revealed negative correlations of the current SCr levels, UPCR levels, and GFR values with the duration after donation, whereas both diastolic and systolic blood pressure correlated positively with the duration after donation. However, none of the renal functions showed statistically significant associations with the duration after donation. A study conducted in 2016 found no severe reduction in the GFR of White kidney donors in long-term follow-up [17]. Likewise, a prospective controlled study found no difference in blood pressure or UPCR levels in kidney donors three years after donation compared with the controls [18].

In terms of comorbidities, we observed that 18.8 percent (n=19) of the patients had hypertension before surgery and that the prevalence of hypertension increased to 31.0 percent (n=31) after surgery. Similarly, a meta-analysis by Boudville et al. concluded that kidney donors may have a 5 mm Hg increase in blood pressure within five to 10 years after donation over the increase anticipated with normal aging [8]. However, Ibrahim et al. found no increased risk of hypertension following kidney donation [5]. We found no association between hypertension and the GFR, consistent with several previous studies [6,7,9-11].

We observed obesity in 11.1% (n=11) of the patients preoperatively and in 12.1% of the patients (n=12) postoperatively. We found no association between BMI and GFR, consistent with the findings of Heimbach [24]. However, Ibrahim et al. reported a statistically significant relationship between GFR and obesity [5].

Limitations

Since the patients were not regularly followed up after donation, we analyzed the data using the current and preoperative values. Hence, a limitation of this study is the lack of a comparison of renal functions at various time intervals (e.g., annually) after donation. Also, missing clinical records may have resulted in the loss of relevant information. Further, we did not analyze such factors as alcohol history, family history of renal disease, or medication history, which can influence renal function.

## Conclusions

In conclusion, we found that GFR values tended to drop immediately postoperatively, but we observed no sustained further drop in GFRs over time. Similarly, we found no statistically significant increase in the risk of developing proteinuria after kidney donation. Older age was the only factor that we found to be related to an increased likelihood of a decrease in the GFR following donor nephrectomy. We observed no statistically significant association between renal function and duration after donation. Clinicians can use these results to reassure donors and encourage live donor kidney transplantation. However, we also recommend that kidney donors comply with the established post-donation follow-up procedures and that initiatives be promoted to increase the level of and enhance clinical follow-up by donors.
